# A modified Delphi study to enhance and gain international consensus on the Physical Activity Messaging Framework (PAMF) and Checklist (PAMC)

**DOI:** 10.1186/s12966-021-01182-z

**Published:** 2021-08-19

**Authors:** Chloë Williamson, Paul Kelly, Jennifer R. Tomasone, Adrian Bauman, Nanette Mutrie, Ailsa Niven, Justin Richards, Graham Baker

**Affiliations:** 1 Physical Activity for Health Research Centre (PAHRC), Institute for Sport, Physical Education and Health Sciences, University of Edinburgh, Edinburgh, UK; 2 School of Kinesiology and Health Studies, Queens University, Kingston, Canada; 3 Sydney School of Public Health, University of Sydney, Sydney, Australia; 4 Faculty of Health, Victoria University Wellington, Wellington, New Zealand; 5 Sport New Zealand Ihi Aotearoa, Wellington, New Zealand

**Keywords:** Exercise, Communication, Guidance, Principles, Consensus

## Abstract

**Introduction:**

Physical activity messaging is an important step in the pathway towards improving population physical activity levels, but best practice is not yet understood. A gap in the literature exists for a physical activity messaging framework to help guide creation and evaluation of messages. This study aimed to further develop and improve, and gain international expert consensus on, a standardised Physical Activity Messaging Framework and Checklist.

**Methods:**

A modified Delphi study consisting of three online survey rounds was conducted. Each survey gathered feedback from an international expert panel using quantitative and qualitative methods. The framework and checklist were amended between each round based on survey results until consensus (defined a priori as 80% agreement) was reached.

**Results:**

The final expert panel (*n* = 40, 55% female) came from nine countries and comprised academics (55%), healthcare and other professionals (22.5%) and government officials or policymakers (22.5%). Consensus was reached in survey 3 with 85 and 87.5% agreement on the framework and checklist, respectively.

**Conclusion:**

This study presents an expert- and evidence-informed framework and checklist for physical activity messaging. If used consistently, the Physical Activity Messaging Framework and Checklist may improve practice by encouraging evidence-based and target audience-focused messages, as well as enhance the research base on physical activity messaging by harmonising key terminologies and improving quality of reporting. Key next steps include further refining the Physical Activity Messaging Framework and Checklist based on their use in real-world settings.

**Supplementary Information:**

The online version contains supplementary material available at 10.1186/s12966-021-01182-z.

## Introduction

Physical inactivity contributes significantly to the global non-communicable disease burden [1] and improving population physical activity (PA) levels will reduce mortality rates [2, 3]. PA messaging, which can be described as “the overall process of creating and delivering PA messages” [4], is an important step in the pathway towards improving population PA levels by targeting individual and social factors such as social norms, perceptions, and awareness of benefits relating to PA [5–7]. However, best practice in PA messaging is not yet understood [4]. A recent scoping review of 123 articles on PA messaging [4] identified four key considerations that formed a rationale for developing a conceptual framework for PA messaging: (i) PA messaging is a complex area of growing interest, (ii) terminologies used for, and understandings of, various PA messaging concepts are inconsistently used, (iii) it is often unclear *how* PA messages will bring about changes in PA behaviour, and (iv) there is limited use of formative evaluation and theory, such as psychological theory or social marketing principles, to inform message development.

Whilst frameworks within the wider field of health communication that provide guidance on developing and evaluating health messages [8] and campaigns [9] do exist, they have not been used widely in PA messaging research. There is also a dearth of application tools to aid translation of such frameworks into practice. To the best of our knowledge, there have been no attempts to date to organise the different concepts that may be considered in PA message development into a usable format. We believe that a consensus-driven messaging framework and accompanying checklist that harmonise understandings of key concepts and encourage PA messages based on theory, formative evaluation and existing evidence would be an important contribution to the field.

A recent scoping review [4] identified a number of concepts relating to three broad overarching area of PA messaging: (i) message aims(s) and pathway(s), (ii) message content, and (iii) message format and delivery. Using these concepts and drawing on existing frameworks and theory [10, 11] we developed and revised a provisional Physical Activity Messaging Framework (PAMF) over the course of a year (March 2019 – April 2020) through consultation with researchers, policymakers and practitioners. Using the provisional PAMF as a starting point, this study aimed to:

1. Further develop and improve a Physical Activity Messaging Framework to guide message creation and evaluation
2. Develop and improve a checklist to accompany the Physical Activity Messaging Framework
3. Gain international expert consensus on the Physical Activity Messaging Framework and Checklist

## Methods

### Study design

A Delphi study is “an iterative process designed to combine expert opinion into group consensus” [12]. A Delphi study was deemed relevant here as it allowed us to seek views from a geographically diverse expert panel and reduced the risk of social conformity associated with other potential study designs, such as focus groups or nominal group technique [13]. This study was a modified Delphi as opposed to a classical Delphi [14] as it did not include an open first round to generate ideas. Rather, the initial idea (our framework) was based on preliminary formative work [4].

### Modified Delphi structure

**Fig. 1:**
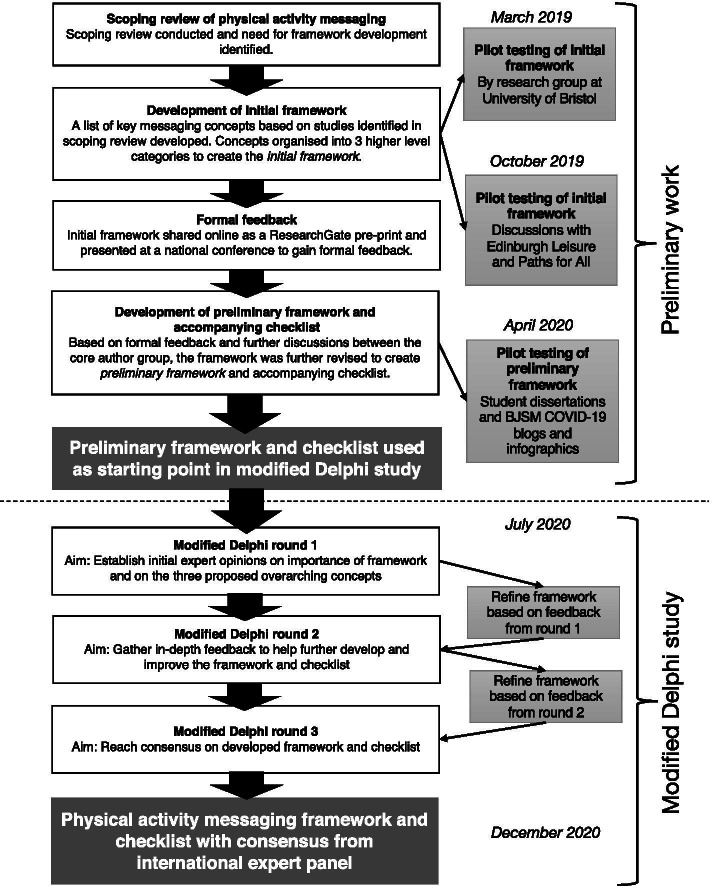
Overview of preliminary work and modified Delphi process

A preliminary framework was developed through formative work and used as a starting point for this study. The modified Delphi process involved international experts participating in three survey rounds, with the framework being amended following each round based on participant feedback. Figure 1 displays an overview of the preliminary work and the modified Delphi process.

### Use of reporting guidelines

We followed the Guidance on Conducting and Reporting Delphi Studies (CREDES) [15].

### Selection and recruitment of expert panel

“PA messaging expert” was defined as ‘an individual within the PA for health field with significant and demonstrable knowledge and/or equivalent experience relevant to PA messaging’. To cover a range of perspectives, we aimed to recruit a heterogenous sample of (i) academics, (ii) healthcare and other professionals, and (iii) government officials and policymakers. We drew on and adapted existing Delphi recruitment guidance [16, 17] to identify participants through active and passive recruitment arms [18]. The active recruitment process involved identifying key disciplines, organisations/groups and literature that we believed would be fruitful in identifying experts before identifying individual experts in each of these areas and inviting them to take part. The passive recruitment process involved advertising the study on Twitter and sending interested individuals an ‘interest to participate form’ to assess their eligibility before inviting them to take part.

### Sample size

Unlike traditional surveys, Delphi studies do not aim to generalise expert opinions. We therefore did not base sample size on achieving statistical power; rather, we aimed to recruit an expert panel that would represent a range of key disciplines and various countries [15]. Delphi literature suggests at least 10–18 expert members per panel are required to achieve a range of opinions [16, 17], and many studies include more than this [19, 20]. We aimed to recruit as many participants as possible to achieve the greatest range of opinions.

### Online surveys

Surveys were delivered online using Qualtrics™ (Qualtrics, Provo, UT). Participants were given 3 weeks to complete each survey round. Participants were sent an initial survey invite via email, and unfinished respondents were sent three reminder emails; the first two generic (“Dear Participant”) and the final reminder personalised (“Dear Name”) to maximise response rate.

### Pilot testing of survey materials

Surveys were pilot tested by nine PA professionals and academics who were not members of the expert panel. This group provided feedback on survey clarity, risk of bias, and suggested improvements. Feedback was collated and discussed by the author group and survey materials amended accordingly.

### Survey 1

Survey 1 aimed to establish initial views on the importance of developing a PA messaging framework and checklist, and on the three proposed overarching concepts (message aim and pathway, message content, and message delivery). Survey 1 collected basic demographic data including professional role, gender, country of residence, and number of years of experience relevant to PA messaging. Participants were also provided with background information to the study and brief descriptions of the three overarching sections of the framework. The full framework and checklist were not shown in survey 1 to avoid overloading participants with information.

Participants were asked to rate the extent to which they agreed or disagreed on a seven-point Likert scale with three statements relating to the importance of developing a PA messaging framework, the proposed overarching concepts, and the usefulness of a checklist tool to accompany the framework. Each Likert-scale question was followed by an open response question where participants had the opportunity to expand. Survey 1 can be found in Additional file 1.

### Survey 2

Survey 2 aimed to gather more in-depth feedback to further refine and improve the (provisional) framework and checklist. Participants were asked to read a summary of survey 1 findings before proceeding. Participants were then shown the framework, which had been updated based on feedback from survey 1, alongside a table of key concepts. Participants then rated the extent to which they agreed or disagreed with a series of statements about seven specific areas of the framework and checklist based on findings from survey 1, such as the role of the framework in aiding message evaluation and terminologies used within the framework. Each seven-point Likert scale question was followed by an open response box, allowing participants to elaborate. Participants were then given an opportunity to provide any other feedback about any of the three overarching framework areas or the checklist. Survey 2 can be found in Additional file 2.

### Survey 3

Survey 3 aimed to either (a) reach consensus on the framework and checklist (updated based on feedback from survey 2), or (b) establish a requirement for a further survey. In survey 3, participants were shown a summary of findings from survey 2 and the updated framework and checklist. Participants then rated the extent to which they agreed or disagreed on a seven-point scale with the following statements: “The Physical Activity Messaging Framework presented here should be the final version” and “The Physical Activity Messaging Checklist presented here should be the final version”, with the opportunity to provide any further feedback on either the framework or checklist. Survey 3 can be found in Additional file 3.

### Defining consensus

Definitions of consensus in Delphi studies vary greatly and are often poorly reported [21]. No universally accepted cut-off for (non)consensus exists. A methodological systematic review of Delphi studies found that cut off for consensus (or non-consensus) was most commonly based on percentage of agreement (usually 75 or 80%), median score or a combination of both. Aligning with CREDES guidance, our definition of consensus was identified a priori as 80% agreement. Specifically, we concluded our Delphi study once > 80% of the expert panel agreed that the framework and checklist presented should be considered final.

### Data analyses

All responses were analysed anonymously and considered equal in weight. Closed questions with Likert scale responses were analysed using descriptive statistics (IBM SPSS Statistics Version 24.0, Armonk, NY) to determine the level of agreement with each statement. We counted the following responses from the seven-point Likert scale as agreement: “*somewhat agree*”, “*agree*” and “*strongly agree*”.

Qualitative data from open responses were analysed using an approach to thematic analysis considered most appropriate for the research aims [22]. The aim of the open questions was to gather in-depth qualitative data which could be used to enhance the framework. We aimed to identify patterns across these data guided by specific areas of the framework, allowing us to identify key aspects that required discussion and further development. Thus, our approach was most consistent with *codebook thematic analysis* [22], involving organic and iterative coding consistent with the broad underlying philosophy of reflexive thematic analysis, but following a more structured approach. In short, this process involved familiarisation with the data, generating codes, and forming themes within pre-determined categories (as informed by the structure of the surveys and areas of the framework). All analyses were conducted by CW with 20% of raw transcripts independently analysed by PK and GB. The authors then took on the roles of ‘critical friends’ [23] where we discussed interpretations of the data, offered alternative interpretations and provided critical feedback, ensuring interpretations were defendable and plausible.

### Framework amendments

Following each survey, the core author team (CW, PK and GB) met to discuss participant feedback. Where feedback within themes was clear enough to allow full interpretation, the framework was amended to address participant views prior to the subsequent survey. Where feedback was not sufficiently clear for us to take action, questions regarding these themes were included in the subsequent survey to further investigate expert opinions and gain more in-depth feedback that could be considered and used to further develop the framework. For each code, our response and any action taken were logged on an Excel sheet.

## Results

### Participants

**Fig. 2:**
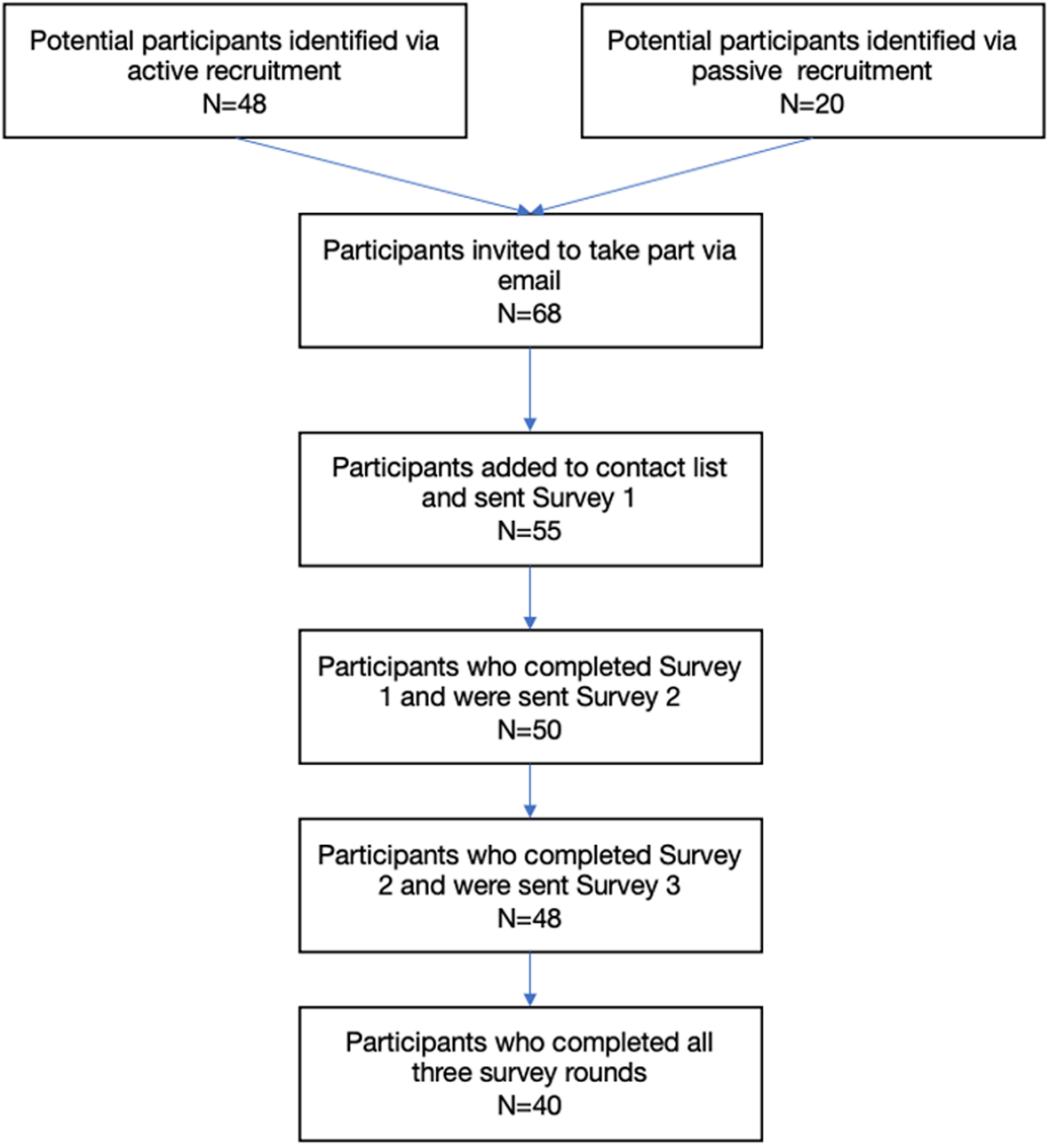
Participant flowchart

A total of 48 experts were identified through active recruitment and a further 20 were identified through the passive recruitment process, resulting in 68 individuals being invited to take part. Of these, 55 (80.8%) agreed to take part via email and were added to the contact list for survey 1. Of these 55, 50 (90.9%) took part in survey 1. Of the participants who took part in Survey 1 (*n* = 50), 48 (96%) completed survey 2, and 40 (80%) completed survey 3. Figure 2 shows the participant flowchart.

The final expert panel (*n* = 40) was 55% female (45% male) and comprised academics (55%), healthcare and other professionals (22.5%), and government officials or policymakers (22.5%). The number of years of experience relevant to PA messaging ranged from 0–1 years to 20 + years, with the majority of participants (65%) reporting 5–20 years of experience. Participants were from nine different countries. The majority of the panel (67.5%, *n* = 27) were recruited via active recruitment, with 13 (32.5%) recruited through passive recruitment. Table 1 shows demographic details of the Delphi study participants in each survey round.

**Table 1:** Demographic characteristics of expert panel for each survey round

	**Survey 1 (*n* = 50)**	**Survey 2 (*n* = 48)**	**Survey 3 (*n* = 40)**
**Gender**			
Male	19 (38%)	19 (39.6%)	18 (45%)
Female	31 (62%)	29 (60.4%)	22 (55%)
**Number of years of experience relevant to physical activity messaging**			
0–1 years	3 (6%)	3 (6.3%)	2 (5%)
2–5 years	8 (16%)	8 (16.6%)	7 (17.5%)
5–10 years	15 (30%)	15 (31.3%)	12 (30%)
10–20 years	18 (36%)	16 (33.3%)	14 (35%)
20 + years	6 (12%)	6 (12.5%)	5 (12.5%)
**Discipline**			
Academia	26 (52%)	25 (52.1%)	22 (55%)
Healthcare professional or other professional	15 (30%)	14 (29.2%)	9 (22.5%)
Government official or policymaker	9 (18%)	9 (18.8%)	9 (22.5%)
**Country of residence**			
Australia	3 (6%)	3 (6.3%)	2 (5%)
Canada	8 (16%)	7 (15%)	6 (15%)
Costa Rica	1 (2%)	1 (2.1%)	1 (2.5%)
India	2 (4%)	2 (4.2%)	0 (0%)
Indonesia	2 (4%)	2 (4.2%)	2 (5%)
Ireland	2 (4%)	2 (4.2%)	2 (5%)
New Zealand	2 (4%)	2 (4.2%)	2 (5%)
Nigeria	1 (2%)	1 (2.1%)	1 (2.5%)
United Kingdom	27 (54%)	26 (54.2%)	22 (55%)
United States	2 (4%)	2 (4.2%)	2 (5%)

### Survey 1

#### Agreement on importance of framework and key concepts included

Of the 50 participants who took part in survey 1, 47 (94%) agreed that establishing a framework for PA messaging is important. There were 46 participants (92%) who agreed that a PA messaging framework should include the following three overarching concepts: “message aims, mechanism and basis”, “message content and format” and “message delivery”. All participants (*n* = 50) agreed that “a checklist tool to accompany the framework would be useful”.

#### Qualitative analysis of open feedback

We identified 28 codes that were organised into 13 themes within the three main areas where feedback was requested (or ‘pre-determined categories’). Key themes highlighted a lack of clarity on the role of the framework in message evaluation, the need to use more plain English to cater for all potential users and the consideration of new concepts such as *language* and *timing*. Survey 1 codes, themes and descriptions can be found in Additional file 4.

#### Subsequent amendments to the framework and checklist

Some minor amendments were made to the framework based on areas where feedback was clear following survey 1. For example, terminologies within the framework were made more user-friendly, such as renaming the heading of section 1 from “Message aim, mechanism and basis” to “What, who, how and why?”. No major amendments were made to the framework at this stage as survey 1 did not collect sufficiently detailed feedback to do so. Indeed, this was not the aim of survey 1. Rather, feedback from survey 1 was used to inform questions in survey 2.

### Survey 2

#### Agreement on key themes that arose in survey 1

Consensus (> 80% agreement) was reached on three of seven Likert scale responses: 39 of the 48 participants (81%) agreed that the “wording/terminology used in the framework is user-friendly and suitable for all potential groups of users of the framework”, 45 participants (94%) agreed that “the concepts within the framework are sufficiently delineated” and that “the checklist meets the aim of being a tool that provides a series of considerations for creating and evaluating PA messages”.

Consensus was not reached in the other four of seven Likert scale questions: 30 participants (62.5%) agreed that “the way the framework could be used to evaluate a message is clear”, 37 participants (77%) agreed that “*language* should be included as a concept within section 2 of the framework”, half (*n* = 24) of the participants agreed that “the promotion of target audience testing is adequately represented in the framework”, and 37 participants (77%) agreed that “*timing* should be included as a concept within the framework”.

#### Qualitative analysis of open feedback

We identified 82 codes that were organised into 45 themes according to the 10 pre-identified categories. Themes identified ranged from minor feedback, such as suggestions to change colours used, to major feedback, such as lack of clarity on how the framework may be used. A full list of themes, codes and example quotes for survey 2 can be found in Additional file 5.

#### Subsequent amendments to the framework and checklist following survey 2

In response to the in-depth feedback from survey 2, a number of amendments were made to improve the framework and checklist. Author responses to each code can be found in Additional file 5. In summary, key changes were made to clarify the framework’s role in various types of evaluation (formative, process, impact/outcome) and to emphasise the importance of engaging with the target audience throughout. We added two dimensions of *language* identified in survey 2; choice of words and message tone, as well as two dimensions of *timing*; time of day and time of year/context. We also added a banner to explicitly encourage consideration of diversity, equity and inclusivity throughout. A number of more minor changes were also made, such as providing further examples in the checklist, adding an arrow to highlight the framework’s pathway, and changes to fonts and colours.

### Survey 3

#### Agreement with final framework and checklist

**Fig. 3:**
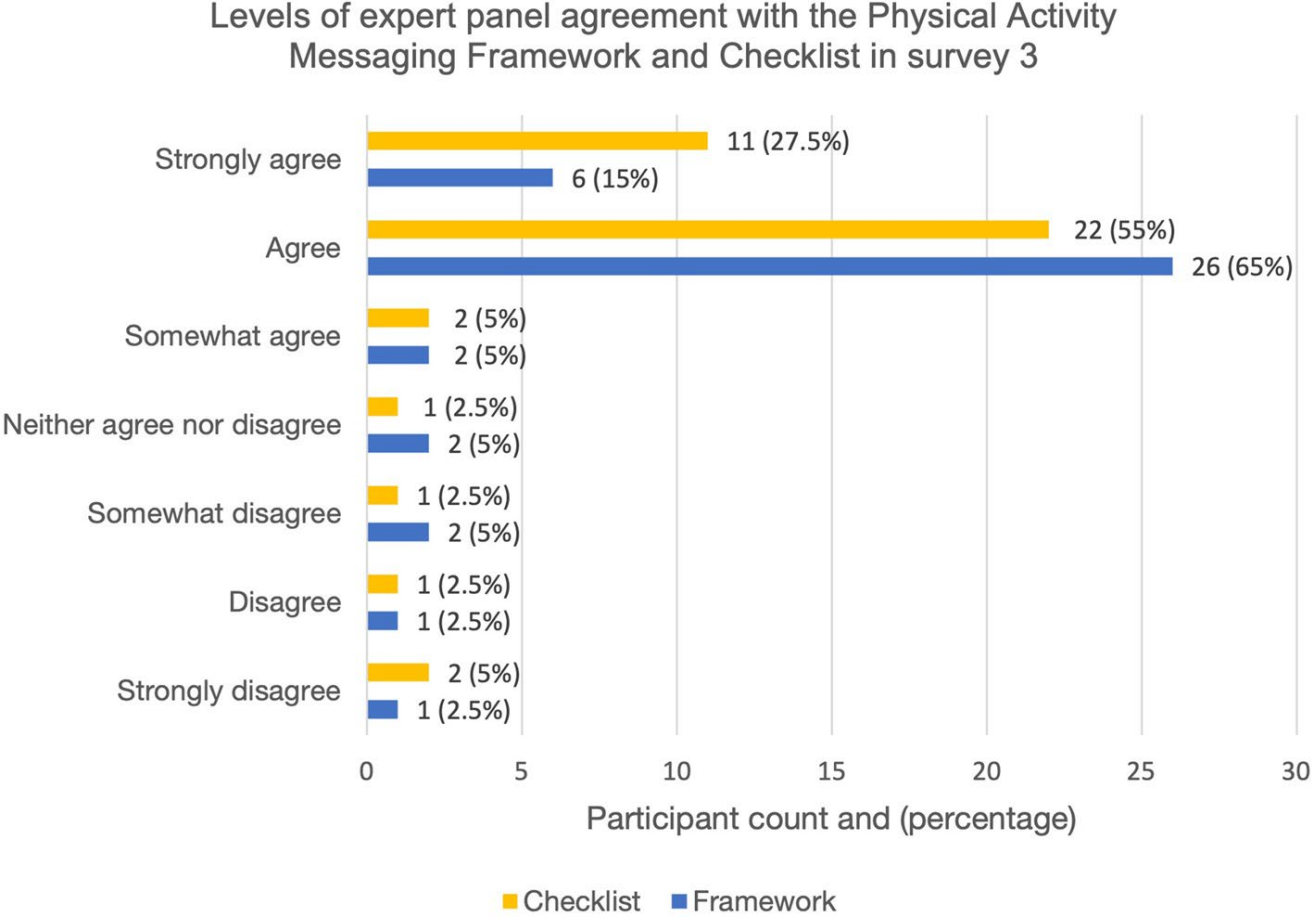
Levels of agreement with the Physical Activity Messaging Framework and Checklist in survey 3

Consensus (defined as > 80% agreement a priori) was reached on both the framework and the checklist in survey 3. Of the final expert panel (*n* = 40), 34 (85%) agreed that the framework presented in survey 3 should be considered final, and 35 (87.5%) agreed that the checklist presented in survey 3 should be considered final. Of these, the majority either *agreed* or *strongly agreed.* Levels of agreement for the Framework and Checklist are displayed in Fig. 3.

#### Qualitative analysis of open response feedback

We identified 40 codes that were organised into 10 themes and categorised into either feedback on (a) the framework or (b) the checklist. The majority of feedback related to minor amendments in wording, visual aspects of the framework and checklist or additions of further explanations and examples. A full list of themes, codes and example quotes for survey 3 can be found in Additional file 6.

### Subsequent amendments to the framework and checklist following survey 3

A small number of minor changes were made to the framework and checklist following survey 3. For example, a title was added, ‘music’ was changed to ‘audio’ to incorporate music, voiceovers and other sounds, and examples of social and political context were added. Author responses to each code can be found in Additional file 6.

### The Physical Activity Messaging Framework and Checklist

**Fig. 4:**
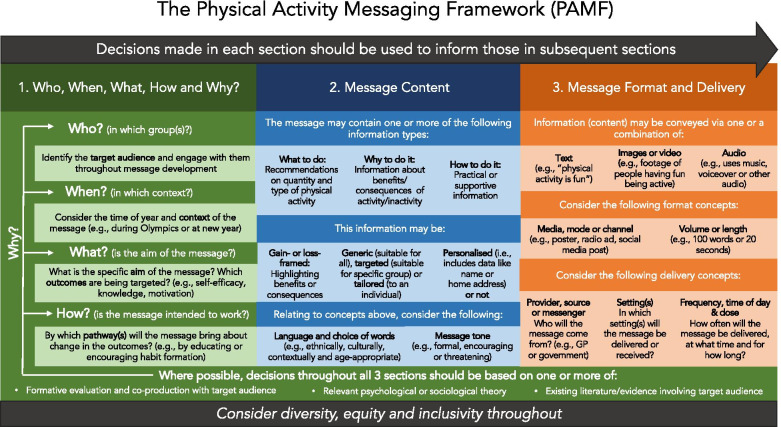
The Physical Activity Messaging Framework (PAMF)

The final agreed framework and checklist are shown in Fig. 4 and Additional file 7, respectively. Working definitions of key concepts within the framework are displayed in Table 2. The PAMF and PAMC are divided into three overarching sections: (1) who, when, what, how and why, (2) message content, and (3) message format and delivery. The PAMF and PAMC are designed to be used sequentially, with decisions in each section being used to inform decisions in subsequent sections. Section 1 encourages the user to identify a target audience, consider the time of year and context of the message, identify specific message aims and potential working pathways, and encourages drawing on psychological theory, formative evaluation and existing evidence to inform message development. Section 2 then guides the user through a series of concepts relating to message content, including the type of information, how this information is framed, and the language and tone of the message. Finally, section 3 encourages the user to consider various concepts relating to the message format and delivery, such as the media or mode of the message, the provider or source of the message and delivery setting. The PAMF provides an overview of messaging concepts for each overarching section and may be a useful visual tool for communications, teaching and training. The PAMC provides a more practical tool for implementing the framework and can be used to guide and document message creation, evaluation, and categorisation. To allow rigour, comprehensive reporting and full transparency, a detailed description of the PAMF and PAMC and how they can be used in practice has been provided in a separate consensus statement and user guide (currently in preparation). This approach is in line with CREDES guidance which encourages publication of a separate paper reporting methodological details if a single publication does not allow for detailed description of both the methods of the Delphi technique and the resulting practice guidance [15].

**Table 2:** Working definitions of key concepts within the framework

**Concept**	**Working definition**	**Example(s)**
**Concepts relating to Section 1: Who, when, what, how and why?**		
**Target audience**	The intended recipient(s) of the message	Older adults, individuals working from home
**Context** [24]	The time of year and the geographical, epidemiological, socio-cultural, socio-economic, ethical, legal and political context at the time of messaging	During the winter, at new year, during a global pandemic
**Outcomes** [25]	Changes expected as a result of messaging	Awareness, understanding, motivation, physical activity behaviour
**Pathway** [25]	The sequential process from the delivery of the message through to outcome. In other words, *how* a message works. This may encompass multiple mechanisms or processes.	Education, persuasion, encouraging habit formation, targeting beliefs about capabilities
**Formative research/evaluation** [26]	Evaluation or research used to help inform message development and to assess whether the message is appropriate and acceptable before it is implemented.	Focus groups with target population to investigate message salience, relevant and importance
**Co-production** (Smith B, Williams O, Bone L. Co-producing research in the sport, physical activity, and exercise sciences: A resource to guide co-production for researchers. Qualitative Research in Sport, Exercise and Health. Forthcoming)	Bringing together citizens with those working in research, policy and industry, and/or practice in an attempt to form equitable partnerships throughout message development	Involving individuals from the target audience in message development
**Concepts relating to Section 2: Message content**		
**‘What to do’ information**	Information regarding the amount or type of physical activity that is recommended	150 min of moderate physical activity per week, 10,000 steps per day, a mixture of aerobic and strength activity
**‘Why to do it’ information**	Information regarding benefits (or consequences) of physical activity (or inactivity)	Physical health, mental health, appearance, environment
**‘How to do it’ information**	Information providing guidance on how to be more active or signposting to opportunities for physical activity	Guidance on when to be active, where to be active or who to be active with
**Use of gain- or loss- framing** [27]	The use of framing a message to highlight either the benefits of taking part in physical activity (gain-framed) or the consequences of not taking part (loss-framed)	Gain-framed: “Walking regularly can make you happier” Loss-framed: “Not walking regularly can increase your risk of depression”
**Tailoring** [28]	Information based on individual user data	Specific feedback on pre-established goals such as step counts
**Targeting** [28]	Information designed to be relevant to a specific group	Information relevant to inactive individuals or people with Diabetes
**Personalisation** [28]	The use of static, user-specific information in a message	Messages involving name or home address
**Language and choice of words**	The dialect(s) and selection of specific wording used in the message	English, Spanish, use of the slang, use of lay-audience friendly language
**Message tone**	The tone adopted by the message	Threatening, persuasive, encouraging
**Concepts relating to Section 3: Message format and delivery**		
**Text (message format)**	The use of words to convey information in a message	Text on posters or social media posts
**Images or video (message format)**	The use of images and videos to convey information in a message	Images or footage of individuals being active
**Audio (message format)**	The use of audio to convey information in a message	Music, voiceovers, sound effects
**Media, mode or channel of delivery**	The type of media through which the message is being communicated	Emails, posters, social media posts, radio/television adverts
**Message volume or length**	The volume or the length of the message relating to the number of words in a message or the amount of time it takes to listen to a message	100 words, 30 s
**Provider or source**	The provider or source of the message	Doctor, journalist, reporter, friends/family
**Setting**	The setting in which the message will be received by the intended recipient	Doctor’s waiting room, home, work
**Frequency, time of day and duration**	How often the message is delivered, at what time, and for how long	Emails sent in the morning 3 times a week for 4 weeks

## Discussion

This study achieved international expert consensus on the PAMF and PAMC from a panel of academics, healthcare and other professionals, and government officials and policymakers. We encourage the use of this evidence- and expert-informed framework and checklist to (a) develop evidence-based PA messages and (b) guide evaluation of such messages. In addition, the PAMC may provide a useful way of understanding and categorising existing messages, for example in evidence reviews.

### Comparisons with and contributions to the literature

The overall process of this modified Delphi was similar to other studies that have sought to gain consensus on a topic informed by preliminary work [19]. This Delphi study had a comparable initial response rate (80.8%) and between-survey response rates (96 and 80% for surveys 2 and 3, respectively) to other similar studies [19, 20]. Consensus was reached in round 3 of the current study. This was expected and is comparable to a number of other Delphi studies that have resulted in frameworks or taxonomies relating to PA or sedentary behaviour, such as a Consensus Taxonomy of Sedentary Behaviours [29], the Comprehensive Analysis of Policy on Physical Activity (CAPPA) framework [30], and a framework for workplace walking and cycling [20].

There are similarities between the PAMF and existing health communication frameworks. The importance of formative research, setting communication objectives and identifying the target audience are also highlighted in the CDC Framework for Health Communication [8, 31]. The importance of considering various types of evaluation (formative, process and outcome) and considering the message itself separately from the message delivery are emphasised in the Audience-Channel-Message-Evaluation (ACME) Framework for Health Communication [9]. To the best of our knowledge, the checklist presented in the current study is the first translational tool specifically designed to assist PA message creation. The agreed PAMF and PAMC have been informed by expert views as well as recent PA messaging literature; an area that has become increasingly researched in the past 5–10 years [4]. The PAMF and PAMC build on existing frameworks by illustrating various PA messaging concepts that could be considered when creating and delivering PA messages, encouraging the user to identify plausible pathways by which the message may bring about changes in outcomes, and by providing a novel checklist tool that can be used to document the process.

### Strengths and limitations

There are a number of study limitations to consider. Firstly, the Delphi method inherently involves subjectivity on a number of levels: in selection of the expert panel, in the experts’ opinions themselves, and in the consideration of feedback and subsequent amendments made to the framework [32]. However, this was deemed the most appropriate method to address the aims of this study and gain feedback from a geographically diverse expert panel. Furthermore, Delphi studies are subject to social conformity bias. We attempted to minimise this bias by analysing data anonymously and by ensuring anonymity between participants. However, we cannot fully eliminate social conformity as the nature of this research involved feeding back opinions from the wider panel to the participants, and we cannot assume that participants were unaware of each other’s involvement in the study.

Although we aimed to capture rich data and feedback from participants through a mixture of quantitative and qualitative approaches, the nature of Delphi studies means opinions may be paraphrased or shortened, and there is always room for misinterpretation [33]. Relatedly, although we reached consensus from an international panel, there are likely insights from others in the wider public health field that would further enhance the framework. Therefore, it may be necessary to further develop and refine the PAMF and PAMC once they have been trialled and tested in applied settings in a variety of disciplinary areas.

Systematic reviews have revealed that almost half (48.8%) of Delphi studies fail to define the percentage threshold for consensus a priori [21], and that only around a third of Delphi studies have international scope [15]. Therefore, key strengths of the current study are that the consensus was defined a priori, and that the framework achieved international consensus. The International Society for Physical Activity and Health (ISPAH) recommends messaging and mass media as a best investment across all countries [6], and the concepts within the PAMF allow flexibility for people in different settings to make contextually relevant decisions. However, despite attempts to broaden inclusion, our expert panel were predominantly from the UK and other high-income countries. This distribution was expected, as our recent scoping review found that 87% of literature on PA messaging came from UK, Canada, USA and Australia [4], however, it does potentially reduce the applicability and usefulness of the PAMF in other countries, particularly low- and middle-income countries. Furthermore, due to our high frequency of UK-based participants, much of the feedback used to develop the PAMF and PAMC came from individuals who may have been more familiar with messaging and campaign attempts in the UK. We therefore invite feedback on and adaption of the PAMF from international colleagues. Despite the limitations, the PAMF and PAMC were developed using rigorous methods and provide a more robust starting point for guidance to inform the creation of PA messages than has previously been available.

### Implications and future directions

The PAMF and PAMC are evidence- and expert-informed tools that allow a range of users to design and evaluate PA messages to any target audience. The PAMF and PAMC have potential to enhance messaging practice by encouraging development of messages based on theory, formative research and existing evidence with emphasis on understanding plausible working pathways and planning appropriate evaluation. Furthermore, we believe that consistent use of the PAMF and PAMC will improve quality of reporting and harmonise understanding of key PA messaging concepts and their definitions, aiding future synthesis and thus understanding of the evidence base.

It is not the purpose of the PAMF and PAMC to provide the user with answers on which decision(s) to make, for example, whether to use gain- or loss-framed messages. Rather, these tools encourage the user to draw on formative research, wider evidence and theory to inform such decisions. Whilst some evidence does exist to support certain decisions over others (e.g., existing evidence is slightly in favour of gain-framed over loss-framed [4, 27]), there is currently not enough evidence to provide recommendations for all relevant concepts within the PAMF. Future research should aim to utilise the PAMF and PAMC to conduct research to develop recommendations for specific messaging concepts in specific population subgroups and contexts.

Improving the functionality and accessibility of the PAMF and PAMC are also key future directions. As an initial next step, we aim to publish a consensus statement and guide to using the framework and checklist to create new messages and aid evaluation and understanding of messages. We see the PAMF and PAMC as iterative and look to continue revising and improving them based on their use in real-world settings. Questions or reports of efforts to employ the PAMF and PAMC can be shared with the corresponding author. We also seek to develop an online interactive tool that will make the PAMC more user-friendly. Finally, we aim to explore the applicability of the PAMF and PAMC in creating and guiding evaluation of messages focused on other health behaviours such as sedentary behaviour.

## Conclusion

PA messaging plays an important role in improving population PA levels. Here, we present a framework and checklist for PA messaging that have consensus from an international expert panel. We believe that the presented framework and checklist, which encourage the design of PA messages based on theory, existing evidence, formative evaluation with the target audience will be an important contribution to our field. If used consistently, the PAMF and PAMC may improve practice by encouraging evidence-based and target audience-focused messages, as well as enhance the research base on PA messaging by harmonising key terminologies and improving quality of reporting.

## Supplementary Information

**Additional file 1.** Survey 1 export.

**Additional file 2.** Survey 2 export.

**Additional file 3.** Survey 3 export.

**Additional file 4.** Survey 1 qualitative analysis.

**Additional file 5.** Survey 2 qualitative analysis.

**Additional file 6.** Survey 3 qualitative analysis.

**Additional file 7.** The PAMC.

## Data Availability

Survey materials and full lists of themes and codes from qualitative data are available in supplementary materials.

## Abbreviations

PA: Physical activity
PAMF: Physical Activity Messaging Framework
PAMC: Physical Activity Messaging Checklist
UK: United Kingdom
USA: United States of America
CMO: Chief Medical Officer
