# Supplementary material for: A modified Delphi study to enhance and gain international consensus on the Physical Activity Messaging Framework (PAMF) and Checklist (PAMC)
Source: Int J Behav Nutr Phys Act. 2021 Aug 19;18:108. doi: 10.1186/s12966-021-01182-z (PMC8375197; doi:10.1186/s12966-021-01182-z)
Supplement: Supplementary file 1 — Additional file 1. Survey 1 export. [file 12966_2021_1182_MOESM1_ESM.docx]

Delphi Survey 1

Start of Block: Information sheet and consent form

Q2 **Information sheet**

**The Physical Activity Messaging framework and checklist: a modified Delphi study**: You are invited to take part in a research study. Before you decide whether to take part or not, it is important for you to understand why the research is being done and what your involvement will consist of. Please take your time to read the following information carefully. Please contact me if there is anything that is unclear to you or that you would like more information on before you decide if you wish to take part.

**What is the purpose of the study?** The aim of this study is to further develop and gain international expert consensus on a conceptual framework for physical activity messaging.

**Why have I been asked to take part?** You have been invited to take part in this study as you have expertise that is relevant to physical activity messaging and we believe you will make a valuable contribution to this study.

**Do I have to take part?** No, you do not have to take part. Participation is entirely voluntary. If you do decide to take part you are free to withdraw at any time by exiting the survey. Deciding not to take part or withdrawing from the study will not affect you negatively in any way. Please note that as responses are anonymous it will not be possible to withdraw your data once you submit the survey.

**What will happen if I do take part?** If you agree to take part, you will be involved in a series (likely 3) of online questionnaires which will each take a maximum of 30 minutes to complete. You will be given 2 weeks to complete each questionnaire, in which time you will be given 2 reminder emails (one in the first week and one in the second week).

**What are the possible benefits of taking part?** If you complete all questionnaires, you will be invited to be included on the author list of the final consensus statement that will be submitted for publication. You will also have contributed to enhancing the area of physical activity messaging research.

**What are the possible disadvantages and risks of taking part?** The Delphi is a time-efficient method and therefore should take less than 2 hours of your time in total. Other than taking up a small amount of your time, we do not think there are any disadvantages of taking part in this study.

**Will my taking part in the study be kept confidential?** Your identity will remain anonymous to other panel members; however, you will be included on the author list of the final published consensus statement if you wish to be. All of the data you provide in the questionnaires will be confidential and it will not be possible to trace statements or opinions from the results back to you.

**What happens when the study is finished?** At the end of the study, we will send you a summary of results for you to read before the final manuscript is published if you wish.

**What will happen to the results of the study?** The results of the study will be included in the principal investigator's PhD thesis. They will be used to write up a consensus statement on the standardised physical activity framework and published in a peer-reviewed journal.

**Who is organising the research and why?** The principal investigator (Chloë Williamson) has been researching this topic through her PhD for almost two years and this study is the next step in developing this work.

**Who has reviewed the study?** The study proposal has been reviewed and accepted by the Moray House School of Education and Sport ethics committee at the University of Edinburgh.

**If you have further questions, please contact:** Chloë Williamson

| Page Break |  |
| --- | --- |

Q31 Informed consent  1. I have read and understood the participant information sheet.

2. I have had an opportunity to ask questions about my participation. 3. I consent for this form to be stored electronically on the University of Edinburgh secure research computer drive. 4. I understand that I am under no obligation to take part in this study. 5. I understand that I have the right to withdraw from this study at any stage without giving any reason. 6. I understand that data collected for the study may be shared with other researchers on an anonymous basis. Data sharing will only be conducted as per the European Union General Data Protection Regulation (2017). Do you agree with the above statements and agree to take part in the study? (Note that if you do not provide consent here and therefore select 'No', you will be taken straight to the end of the survey) 

- Yes (1)
- No (2)

| Page Break |  |
| --- | --- |

Q32 Please provide your job title/professional role in the box below

________________________________________________________________

Q35 Please select the option that best describes your role

- Academic (1)
- Healthcare professional or other professional (2)
- Government official or policymaker (3)

Q36 Please provide your country of residence

________________________________________________________________

Q37 What is your gender?

- Female (1)
- Male (2)
- Non-binary (3)
- Prefer not to say (4)
- Prefer to self-describe (5) ________________________________________________

Q38 Please estimate the number of years of experience you have that are relevant to physical activity messaging

- 0-1 years (1)
- 2-5 years (2)
- 5-10 years (3)
- 10-20 years (4)
- 20+ years (5)

| Page Break |  |
| --- | --- |

Q39 Study background information
 
The overall aim of this study is to develop, and gain international expert consensus/agreement on, a standardised framework for designing and evaluating physical activity messages. In other words, we are not aiming to create PA messages themselves but rather a set of principles/guidance that can be used to help develop and evaluate PA messages. Based on our research to date, we have developed a draft of the Physical Activity Messaging Framework (PAMF), and have used this as a starting point in this study. This study will take the form of a series of surveys (likely 3 in total) that ask questions about the developed framework, with each survey building on the last. 
 

Effective physical activity (PA) messaging may play a role in increasing population PA levels. We have defined PA messaging as a subtype of health communication and as an overall concept encompassing both content and delivery aspects of a PA message. These definitions were developed for the purpose of this research, drawing on existing literature.

| Term | Working definition |
| --- | --- |
| Physical activity messaging | The overall process of designing, creating and delivering physical activity messages **for and to the public** |
| Physical activity message | Educational or persuasive material to be relayed to a specific individual or group **within the public** with the aim of ultimately increasing physical activity levels |
| Physical activity message content | The specific aspects which comprise a PA message, such as the type, amount and presentation of information |
| Physical activity message delivery | The process by which a physical activity message is delivered to the target individual or group of the public |

 

Q40 Click to write the question text

| Page Break |  |
| --- | --- |

Q41 We conducted a scoping review of PA messaging that was published earlier this year (Williamson et al., 2020). This scoping review revealed 3 key considerations.   1.     Firstly, PA messaging is a complex and multidimensional area and best practice is not yet well understood. To the best of our knowledge, there have been no attempts to date to organise or categorise the different components within PA messaging.   2.     Secondly, terminologies used for and understandings of the various PA messaging concepts are inconsistent across the literature. This makes it difficult to summarise and collate what is known about different concepts.   3.     Finally, it is often unclear what the proposed mechanism(s) are by which PA messaging interventions aim to bring about changes in PA, i.e. “how” they are expected to work. Relating to this, our scoping review found that many studies did not draw on or failed to adequately report use of formative research, psychological theory or social marketing principles to guide messages creation.

| Page Break |  |
| --- | --- |

Q42 We believe a framework that organises PA messaging concepts, harmonises terminologies relating to PA messaging, and encourages the design of messages based on theory, formative work and existing evidence with emphasis on understanding plausible mechanisms of action would be an important contribution to the field. Using the concepts identified in the scoping review and drawing on existing psychological theory, a conceptual framework for PA messaging and an accompanying checklist have been developed.

| Page Break |  |
| --- | --- |

Q43  The specific aim of this first survey is to understand overall expert opinions on the need for and importance of a physical activity messaging framework, as well as to gather high level feedback on the overarching concepts within the framework. Please read the following information on the **PA messaging framework** (PAMF) and **checklist** (PAMC) before responding to the survey questions.

| Page Break |  |
| --- | --- |

Q45 **The physical activity messaging framework (PAMF)** The full framework as it currently stands will be shared in the next survey round.
The developed framework has 3 high level categories which each encompass multiple sub-concepts. Further detail on the sub-concepts will be provided in the next survey round. Descriptions of the 3 high level categories within the framework are below: *1.     Message aim, mechanism and basis*This section encourages establishing a clear aim by encouraging consideration of the target population, as well as the mechanism(s) by which the message is intended to work (e.g. which specific determinants are being targeted) and what such decisions are being based on (such as drawing on psychological theory or formative research). *2.     Content and format*This section encourages consideration of the type of information that is used in the message; framing of this information; tailoring, targeting or personalisation of the message; the use of text, images, video and music; the media/mode of the message and the volume and length of the message.*3.     Delivery*This section encourages consideration of who will deliver the message, where the message will be delivered, who the message will be delivered to and the frequency and dose of delivery.

Q25

Q23 Click to write the question text

| Page Break |  |
| --- | --- |

Q44 The physical activity messaging checklist (PAMC) A practical checklist to accompany the PAMF has been created for use in both message creation and evaluation. This checklist aims to provide a practical tool that academics, practitioners and other relevant stakeholders can use to work their way through the 3 key overarching concepts outlined above and (as appropriate) consider each concept when (a) creating a message or (b) evaluating a message. In message creation, the checklist encourages the message creator to consider (and document this consideration) for each relevant item within the framework. In message evaluation, the checklist provides a tool for categorising and understanding existing physical activity messages - guiding process, impact and outcome evaluation.

End of Block: Information sheet and consent form

Start of Block: Survey section 1

Q9 When answering the following questions, please consider the creation and evaluation of **physical activity messages for the public**.

Q10 Q1. Please rate the extent to which you agree with the following statement.

|  | Strongly disagree (1) | Disagree (2) | Somewhat disagree (3) | Neither agree nor disagree (4) | Somewhat agree (5) | Agree (6) | Strongly agree (7) |
| --- | --- | --- | --- | --- | --- | --- | --- |
| Establishing a framework for physical activity messaging is important. (1) |  |  |  |  |  |  |  |

| Page Break |  |
| --- | --- |

Q15 Q2. If you do not agree that establishing a framework for physical activity messaging is important, could you please say a little more about your thoughts around this.

________________________________________________________________

| Page Break |  |
| --- | --- |

Q13 Q3. Please rate the extent to which you agree with the following statement.

|  | Strongly disagree (1) | Disagree (2) | Somewhat disagree (3) | Neither agree nor disagree (4) | Somewhat agree (5) | Agree (6) | Strongly agree (7) |
| --- | --- | --- | --- | --- | --- | --- | --- |
| A physical activity messaging framework should include the following 3 overarching concepts: “message aims, mechanism and basis”, “message content and format” and “message delivery”. (1) |  |  |  |  |  |  |  |

| Page Break |  |
| --- | --- |

Q17 Q4. If you do not agree with the 3 overarching concepts outlined in the question above, or the suggestion to have 3 overarching concepts, could you please say a little more on your thoughts around this.

________________________________________________________________

| Page Break |  |
| --- | --- |

Q14 Q5. Please feel free to suggest names and definitions of any additional and/or alternative overarching concepts and briefly explain why you think they should be added or amended.

________________________________________________________________

| Page Break |  |
| --- | --- |

Q18 Q6. Please rate the extent to which you agree with the following statement.

|  | Strongly disagree (1) | Disagree (2) | Somewhat disagree (3) | Neither agree nor disagree (4) | Somewhat agree (5) | Agree (6) | Strongly agree (7) |
| --- | --- | --- | --- | --- | --- | --- | --- |
| A checklist tool to accompany the framework would be useful for those who are creating or evaluating physical activity messages. (1) |  |  |  |  |  |  |  |

| Page Break |  |
| --- | --- |

Q19 Q7. If you do not agree that a checklist tool to accompany the framework would be useful, please feel free to say why.

________________________________________________________________

| Page Break |  |
| --- | --- |

End of Block: Survey section 1
