# Supplementary material for: A modified Delphi study to enhance and gain international consensus on the Physical Activity Messaging Framework (PAMF) and Checklist (PAMC)
Source: Int J Behav Nutr Phys Act. 2021 Aug 19;18:108. doi: 10.1186/s12966-021-01182-z (PMC8375197; doi:10.1186/s12966-021-01182-z)
Supplement: Supplementary file 2 — Additional file 2. Survey 2 export. [file 12966_2021_1182_MOESM2_ESM.docx]

Delphi Survey 2

Start of Block: Default Question Block

 
Thank you again for taking part in this *Physical Activity Messaging Delphi study*, and thank you for your responses to Survey 1. Your time and expert opinions are much appreciated. 
 
Please remember that you can withdraw from this study at any time. 
 
**Please click the link below and read the summary of findings from Survey 1 before proceeding.**

Click here to open summary of survey 1 findings.

| Page Break |  |
| --- | --- |

Based on results from Survey 1, we have amended some aspects of the framework. Other aspects that we received feedback on are presented in this survey to gain further insight from the panel. **Please click the link below to open the current version of the framework** and table of key concepts in a separate window to refer to it throughout the course of this survey.

Click here to open the framework.

| Page Break |  |
| --- | --- |

Q1. It was proposed in Survey 1 that 'evaluation' should be added to or included in the framework. This framework is intended to be used as an evaluation tool itself. Having now seen the full framework, please rate the extent to which you agree or disagree with the following statement:

|  | Strongly disagree (1) | Disagree (2) | Somewhat disagree (3) | Neither agree nor disagree (4) | Somewhat agree (5) | Agree (6) | Strongly agree (7) |
| --- | --- | --- | --- | --- | --- | --- | --- |
| The way that the framework could be used to evaluate a message is clear. |  |  |  |  |  |  |  |

Q2. Please use the box below to provide any feedback on the use of the framework as an evaluation tool.

________________________________________________________________

| Page Break |  |
| --- | --- |

Q3. It was proposed in Survey 1 that 'language' should be included as a concept within section 2 of the framework (with 'language' referring to the use of language types that may be used in messages such as forceful, informal, professional, age-appropriate etc). Please rate the extent to which you agree or disagree with the following statement:

|  | Strongly disagree (1) | Disagree (2) | Somewhat disagree (3) | Neither agree nor disagree (4) | Somewhat agree (5) | Agree (6) | Strongly agree (7) |
| --- | --- | --- | --- | --- | --- | --- | --- |
| Language should be included as a concept within the framework |  |  |  |  |  |  |  |

Q4. Please use the box below to provide any feedback on the inclusion of 'language' as a concept within the framework.

________________________________________________________________

| Page Break |  |
| --- | --- |

Q5. Survey 1 findings suggested that the framework should use more plain English so that it is user-friendly for all potential groups of users of the framework (academics, healthcare professionals, other professionals and government officials or policymakers). As a result we have changed some of the wordings within the framework and have provided examples for some concepts. Please rate the extent to which you agree or disagree with the following statement:

|  | Strongly disagree (1) | Disagree (2) | Somewhat disagree (3) | Neither agree nor disagree (4) | Somewhat agree (5) | Agree (6) | Strongly agree (7) |
| --- | --- | --- | --- | --- | --- | --- | --- |
| The wording/terminology used in the framework is user-friendly and suitable for all potential groups of users of the framework |  |  |  |  |  |  |  |

Q6. Please use the box below to provide any feedback on the wording/terminology used in the framework.

________________________________________________________________

| Page Break |  |
| --- | --- |

Q7. Survey 1 findings suggested that the various concepts within the framework should be delineated (i.e. concepts should be more clearly outlined and defined). We have attempted to do this by numbering concepts and by using visual tools such as colour. Please rate the extent to which you agree or disagree with the following statement.

|  | Strongly disagree (1) | Disagree (2) | Somewhat disagree (3) | Neither agree nor disagree (4) | Somewhat agree (5) | Agree (6) | Strongly agree (7) |
| --- | --- | --- | --- | --- | --- | --- | --- |
| The concepts within the framework are sufficiently delineated. |  |  |  |  |  |  |  |

Q8. Please use the box below to provide any feedback on the clarity of individual concepts within the framework.

________________________________________________________________

| Page Break |  |
| --- | --- |

Q9. The results from Survey 1 suggested that research with the target audience to help inform message creation should be encouraged by the framework. We believe this is reflected in Section 1 of the framework. Having now seen the full framework, please rate the extent to which you agree or disagree with the following statement.

|  | Strongly disagree (1) | Disagree (2) | Somewhat disagree (3) | Neither agree nor disagree (4) | Somewhat agree (5) | Agree (6) | Strongly agree (7) |
| --- | --- | --- | --- | --- | --- | --- | --- |
| The promotion of target audience testing is adequately represented in the framework. |  |  |  |  |  |  |  |

Q10. Please use the box below to provide any feedback on how well the framework encourages audience testing.

________________________________________________________________

| Page Break |  |
| --- | --- |

Q11. Findings from Survey 1 suggested that 'timing' may be an important concept to include alongside message 'frequency and dose' in section 3 of the framework. Please rate the extent to which you agree or disagree with the following statement:

|  | Strongly disagree (1) | Disagree (2) | Somewhat disagree (3) | Neither agree nor disagree (4) | Somewhat agree (5) | Agree (6) | Strongly agree (7) |
| --- | --- | --- | --- | --- | --- | --- | --- |
| Timing should be included as a concept within the framework alongside message frequency and dose. |  |  |  |  |  |  |  |

Q12. Please use the box below to provide any feedback on the inclusion of 'timing' as a concept within the framework.

________________________________________________________________

| Page Break |  |
| --- | --- |

Survey 1 results showed that 92% of the panel members agreed with the three proposed overarching concepts of the framework. Having now seen the full framework and reviewed each concept, do you have any specific feedback on:

Q13. Section 1 of the framework: "Who, how and why"? (please use the box below)

________________________________________________________________

Q14. Section 2 of the framework: "What is the message"? (please use the box below)

________________________________________________________________

Q15. Section 3 of the framework: "How is the message delivered"? (please use the box below)

________________________________________________________________

| Page Break |  |
| --- | --- |

In addition to the Physical Activity Messaging Framework (PAMF), we have created a checklist that can be used as a practical tool to document the process of message creation or evaluation using the framework. Survey 1 results suggested that 100% of participants agreed this would be useful. **Please click the link below to open the checklist** in a separate window before proceeding.

Click here to open the checklist

Q16. Survey 1 findings showed that participants believed a checklist should not be a prescriptive list, but rather should provide considerations for messaging. This was our aim when creating the checklist. Having now seen the checklist, please rate the extent to which you agree or disagree with the following statement.

|  | Strongly disagree (1) | Disagree (2) | Somewhat disagree (3) | Neither agree nor disagree (4) | Somewhat agree (5) | Agree (6) | Strongly agree (7) |
| --- | --- | --- | --- | --- | --- | --- | --- |
| The checklist meets the aim of being a tool which provides series of considerations for creating or evaluating physical activity messages |  |  |  |  |  |  |  |

Q17. Please use the box below to provide any other feedback on any aspect of the checklist.

________________________________________________________________

| Page Break |  |
| --- | --- |

Please use this opportunity to go back and amend/complete any answers if you need to. Proceeding to the next page will end the survey at which point you cannot go back.

End of Block: Default Question Block
