# Supplementary material for: A modified Delphi study to enhance and gain international consensus on the Physical Activity Messaging Framework (PAMF) and Checklist (PAMC)
Source: Int J Behav Nutr Phys Act. 2021 Aug 19;18:108. doi: 10.1186/s12966-021-01182-z (PMC8375197; doi:10.1186/s12966-021-01182-z)
Supplement: Supplementary file 3 — Additional file 3. Survey 3 export. [file 12966_2021_1182_MOESM3_ESM.docx]

Survey 3

Start of Block: Default Question Block

Q1 Thank you again for taking part in this Physical Activity Messaging Delphi study, and thank you for your responses to Survey 2. Your time and expert opinions are much appreciated.  
   
 Please remember that you can withdraw from this study at any time.  
 
The results of this survey will determine whether or not there is a subsequent survey. 
    

If you have any questions at any point please do not hesitate to contact me.
 
 
Structure of Survey
  This survey will take you approximately 10-15 minutes to complete. This survey will firstly provide you with the opportunity to see the results from Survey 2 and the updated Framework and Checklist. 
 
The survey will then ask a total of **4 questions**: 
- 2 questions relating to the Physical Activity Messaging FRAMEWORK 
- 2 questions relating to the Physical Activity Messaging CHECKLIST

| Page Break |  |
| --- | --- |

Q23 Please use the link below to view the updated Framework and Checklist before proceeding.

Q24 Click to write the question text

Q26 Further detail on the Framework and Checklist can be found in the article below. This is a first draft of the article that will accompany the Framework and Checklist. Reading this article is **entirely optional** and not essential for completing this survey.

Q27 Click to write the question text

Q25 I have viewed the updated Framework and Checklist and I am ready to proceed.

- Yes (1)

| Page Break |  |
| --- | --- |

Q14 **Findings from Survey 2**
 
*Please read the below summary of findings from Survey 2 before proceeding. A link to a full version of results is available at the end of this page.*

 Likert scale responses 
  
Of the 50 participants who completed Survey 1, 48 completed Survey 2 (96% response rate). 
 
Participants responded to 7 Likert scale questions in Survey 2. Consensus (>80% agreement) was reached on 3 out of these 7: 
 ·       39 of the participants (81%) agreed (either somewhat agreed, agreed or strongly agreed) that the wording/terminology used in the framework is user-friendly and suitable for all potential groups of users of the framework. ·       45 of the 48 participants (94%) agreed that the concepts within the framework are sufficiently delineated. ·       Finally, 45 (94%) of participants agreed that the checklist meets the aim of being a tool which provides a series of considerations for creating and evaluating physical activity messages.   Consensus was not reached in the other 4 out of 7 Likert scale questions: ·       30 participants (62.5%) agreed that the way the framework could be used to evaluate a message is clear. ·       37 of the 48 (77%) agreed that ‘language’ should be included as a concept within section 2 of the framework. ·       Half (n=24, 50%) of the participants agreed that the promotion of target audience testing is adequately represented in the framework. ·       37 (77%) of the participants agreed that timing should be included as a concept within the framework alongside message frequency and dose.


 Analysis of qualitative feedback and subsequent amendments to the Framework and Checklist We also analysed over 10,000 words of qualitative feedback from Survey 2. We identified a number of themes that resulted in amendments to the Framework and Checklist. A summary of key amendments and responses are below:  
 In response to feedback on the framework as an evaluation tool: We see the primary purpose of the framework being to aid the creation of new messages, and we encourage the use of formative evaluation guided by the concepts in the framework in such research. However, we also hope that the framework may be useful in planning process and impact/outcome evaluation by helping to identify message aims to inform evaluation and indicators that could be measured. We have now clearly defined the different types of evaluation (formative, process and outcome/impact) in our accompanying writing piece and have provided guidance on how the framework may play a role in the different types of evaluation. We have added a ‘what’ category in Section 1 of the framework that encourages the user to identify specific aims and outcomes of the message. We hope that this will be helpful for users when planning outcome/impact evaluation.
 In response to feedback on the clarity of involving the target audience We have amended the wording of ‘consider the target audience’ to ‘engage with’ We have added in ‘co-production’ to make it clearer that this aligns with formative evaluation (encouraged in Section 1) We have updated wording around drawing on existing evidence so it is clear this should be evidence regarding the target audience We have extended the “why” section to make it clearer that these considerations should inform decisions throughout all 3 sections of the framework
 In response to feedback on including ‘language’ as a concept: Participant feedback highlighted 2 key dimensions of language: (1) appropriate language and choice of words relating to ethnicity, nationality, age, culture and context, and (2) the tone of the message. We have now included these in the framework in Section 2 (content and format).
 In response to feedback on including ‘timing’ as a concept: Participant feedback highlighted 3 types or dimensions of ‘timing’ and we have made the follow changes to address these: Time of day has been added alongside ‘frequency and dose’ in section 3 of the framework Time of year/context has been incorporated by adding a ‘when’ box in section 1 of the framework Timing in relation to psychological readiness will be highlighted as a potential area of focus in message targeting and tailoring in the accompanying writing piece
 Other amendments We have provided more examples for various concepts in both the framework and checklist and will expand on this in the accompanying writing. We have added an arrow to make pathway through framework clearer We have changed the colour of section 1 from grey to green We have harmonised format of the three sections by removing the numbering system from section 1 and by adding subheadings to section 3 We have moved the formatting concepts from section 2 to 3 We have amended “tick **all** that apply” in the checklist to “tick **those** that apply” and have added a note to the checklist preamble that the framework and checklist outline available dimensions/concepts rather than encourage the use of all (i.e. more ticks does not mean better message). We have added a banner to the framework to encourage consideration of and commitment to diversity, equity and inclusion throughout the different concepts of the framework. We have included many of the themes that resulted from participant feedback as discussion points in the accompanying writing piece to the PAMF and PAMC. *Please click the link below if you would like to read the full version of the Survey 2 results before proceeding with this survey.*

Q2 Click to write the question text

Q15 I have read either the summary or full version of results from Survey 2 and am ready to proceed.

- Yes (1)

| Page Break |  |
| --- | --- |

Q11 Please respond to the following questions regarding the Physical Activity Messaging **Framework**

Q16

Q5 1. Please rate the extent to which you agree or disagree with the following statement:

|  | Strongly disagree (1) | Disagree (2) | Somewhat disagree (3) | Neither agree nor disagree (4) | Somewhat agree (5) | Agree (6) | Strongly agree (7) |
| --- | --- | --- | --- | --- | --- | --- | --- |
| The Physical Activity Messaging Framework (PAMF) presented here should be the final version. (1) |  |  |  |  |  |  |  |

Q7 2. Please use the box below to give any further feedback on the Physical Activity Messaging **Framework**.

________________________________________________________________

| Page Break |  |
| --- | --- |

Q12 Please respond to the following questions regarding the Physical Activity Messaging **Checklist**

Q18 Click to write the question text

Q6 3. Please rate the extent to which you agree or disagree with the following statement:

|  | Strongly disagree (1) | Disagree (2) | Somewhat disagree (3) | Neither agree nor disagree (4) | Somewhat agree (5) | Agree (6) | Strongly agree (7) |
| --- | --- | --- | --- | --- | --- | --- | --- |
| The Physical Activity Messaging Checklist (PAMC) presented in this survey should be the final version. (1) |  |  |  |  |  |  |  |

Q9 4. Please use the box below to give any further feedback on the Physical Activity Messaging **Checklist**.

________________________________________________________________

| Page Break |  |
| --- | --- |

Q13 Please use this opportunity to go back and revisit any questions in the survey. Proceeding to the next page will end the survey and you will be unable to go back.

Q19 I am happy with my responses and ready to proceed to the end of the survey.

- Yes (1)

End of Block: Default Question Block
