# Supplementary material for: A modified Delphi study to enhance and gain international consensus on the Physical Activity Messaging Framework (PAMF) and Checklist (PAMC)
Source: Int J Behav Nutr Phys Act. 2021 Aug 19;18:108. doi: 10.1186/s12966-021-01182-z (PMC8375197; doi:10.1186/s12966-021-01182-z)
Supplement: Supplementary file 7 — Additional file 7. The PAMC. [file 12966_2021_1182_MOESM7_ESM.docx]

| **Physical Activity Messaging Checklist (PAMC)** | | | |
| --- | --- | --- | --- |
| This checklist has been designed to be used in conjunction with the Physical Activity Messaging Framework (PAMF).  **When aiming to create new messages:** The user is encouraged to work sequentially through the checklist, with the decisions made in Section 1 informing subsequent sections and using the checklist as a reporting framework. This checklist is not a prescriptive set of instructions but rather a set of considerations for creating physical activity messages. The concepts in this checklist may also be used to guide formative research/evaluation with the target audience which in turn can be used to make decisions around message content and delivery.  **When aiming to evaluate or understand existing messages:** The checklist may be used to plan a process, impact or outcome evaluation. It can help identify message aims to inform evaluation and indicators that could be measured. It can also help to understand existing messages by allowing the user to classify messages and identify potential effective message components.  The ‘tick those that apply’ column is designed to aid you in keeping track of which concepts have been considered and how and what decisions were made, not to necessarily encourage use of all concepts. A message with more ticks is not by default better than a message with fewer ticks. | | | |
| **Reason for using checklist** | Tick those that apply | Additional comments/description | |
| I am using this checklist to create a new message |  |  | |
| I am using this checklist to understand an existing message, or to inform process and/or impact/outcome evaluation of an existing message |  |  | |
| 1. **Who, When, What, How and Why?** | | | |
| - 1. Who? (in which group(s)?) | | | |
|  | Tick those that apply | | Specify below |
| - - 1. Target population identified |  | |  |
| - - 1. Target audience engaged with to inform message development |  | |  |
| - 1. When? (in which context?) |  | |  |
|  | Tick those that apply | | Specify below |
| - - 1. Time of year and social/political context of message considered (e.g., during the Olympics or during the COVID-19 pandemic) |  | |  |
| - 1. What? (is the aim of the message?) |  | |  |
|  | Tick those that apply | | Specify below |
| - - 1. Specific aim of message identified (e.g., to improve self-efficacy, motivation, awareness, perceptions, knowledge etc) and specific outcomes relating to the aim identified and clearly stated |  | |  |
| - 1. How? (is the message intended to work?) | | | |
|  | Tick those that apply | | Specify below |
| - - 1. Potential pathway(s) by which message may bring about change in the outcome(s) of interest identified (e.g., targeting beliefs about capabilities) |  | |  |
| - 1. Why? (are the decisions around message creation being made?) | | | |
|  | Tick those that apply | | Specify below |
| - - 1. Decisions based on psychological or sociological theory or social marketing principles |  | |  |
| - - 1. Decisions based on formative evaluation or co-production with the target audience (note: the concepts in this Checklist can be used to inform areas of investigation in research with the target audience) |  | |  |
| - - 1. Decisions based on existing literature and evidence involving the target audience |  | |  |
| 1. **Message Content (what is in the message?)** | | | |
| - 1. Type of information | | | |
|  | Tick those that apply | | Additional comments/description |
| - - 1. Message contains “what to do” information (quantity and type of activity). For example: “Aim for 10,000 steps a day!” |  | |  |
| - - 1. Message contains “why you should do it” information (e.g., physical, mental, social health, environmental benefits or appearance-based information). For example: “Take the stairs – feel less stressed!” |  | |  |
| - - 1. Message contains “how to do it” information (practical or supportive information). For example: “Did you know that we run a group walk for older adults every Thursday at 12pm?” |  | |  |
| - 1. Information framing, targeting, tailoring and personalisation | | | |
|  | Tick those that apply | | Additional comments/description |
| - - 1. Message content is gain-framed (highlights benefits) |  | |  |
| - - 1. Message content is loss-framed (highlights consequences) |  | |  |
| - - 1. Message content is generic (suitable for all) |  | |  |
| - - 1. Message content is tailored to an *individual* (based on user-specific data such as personal step count goal) |  | |  |
| - - 1. Message content is *targeted* at a group (e.g., Type 2 diabetics or inactive older adults) |  | |  |
| - - 1. Message content is *personalised* (contains personal information such as name or home address) |  | |  |
| - 1. Use of language | | | |
|  | Tick those that apply | | Additional comments/description |
| - - 1. Appropriate language and choice of words considered (e.g., ethnically, culturally, contextually and age-appropriate) |  | |  |
| - - 1. Message conveyed using a particular tone (e.g., formal, encouraging or threatening) |  | |  |
| 1. **Message Format and Delivery** | | | |
| - 1. The way the information (content) is conveyed | | | |
|  | Tick those that apply | | Additional comments/description |
| - - 1. Message uses text to convey information (e.g., “physical activity is fun”) |  | |  |
| - - 1. Message uses images or videos to convey information (e.g., images or footage of people having fun being physically active) |  | |  |
| - - 1. Message uses music to convey information (e.g., the use of ‘fun’ music in the message) |  | |  |
| - 1. Message format |  | |  |
|  | Tick those that apply | | Additional comments/description |
| - - 1. The media, mode or channel of the message has been considered/specified |  | |  |
| - - - 1. *Radio advert* |  | |  |
| - - - 1. *TV advert* |  | |  |
| - - - 1. *Poster* |  | |  |
| - - - 1. *Leaflet or pamphlet* |  | |  |
| - - - 1. *Social media post (specify platform e.g., Twitter, Facebook, Instagram, TikTok, Snapchat etc)* |  | |  |
| - - - 1. *Email* |  | |  |
| - - - 1. *SMS/text message* |  | |  |
| - - - 1. *Other* |  | |  |
| - - 1. The message is of a specified length or volume (e.g., 100 words or 20 seconds) |  | |  |
| - 1. Message delivery |  | |  |
|  | Tick those that apply | | Additional comments/description |
| 3.3.1. The provider, messenger or source has been considered/specified |  | |  |
| - - 1. *Health care professional (e.g., GP)* |  | |  |
| - - 1. *Family or friends* |  | |  |
| - - 1. *Peers* |  | |  |
| - - 1. *The media* |  | |  |
| - - 1. *The Government* |  | |  |
| - - 1. *Celebrities* |  | |  |
| - - 1. *Other (specify in additional comments/description box)* |  | |  |
| - 1. The setting in which the message will be delivered has been considered and specified, e.g., at home, at school, at work, at the doctor’s surgery, at bus stops etc. |  | |  |
| - 1. The frequency, time of day and dose of message delivery have been considered and specified, e.g., 3 messages a week, set at 9am, for 3 months. |  | |  |
